# The Role of Telemedicine in Enhancing Chronic Kidney Disease (CKD) Management and Dialysis Care

**DOI:** 10.7759/cureus.55816

**Published:** 2024-03-08

**Authors:** Ramchandani Santosh, Yaqub Nadeem Mohammed, Zubair Rahaman, Sakshi Khurana

**Affiliations:** 1 Internal Medicine/Nephrology, Lehigh Valley Hospital, Allentown, USA; 2 Internal Medicine, Western Michigan University Homer Stryker M.D. School of Medicine, Kalamazoo, USA; 3 Internal Medicine, University at Buffalo Jacobs School of Medicine and Biomedical Sciences, Buffalo, USA; 4 Radiology, New York Presbyterian/Columbia University Irving Medical Center, New York, USA

**Keywords:** chronic kidney disease (ckd), end-stage kidney disease (eskd), dialysis care, video telemedicine, telemedicine (tm)

## Abstract

Telemedicine has emerged as a transformative solution in the realm of healthcare, particularly in addressing the complexities and challenges associated with chronic kidney disease (CKD) and dialysis care. This editorial explores the potential of telemedicine in revolutionizing the management and treatment of kidney diseases, highlighting its role in mitigating the burdens faced by healthcare systems worldwide. With the advent of high-quality audio and visual platforms, telemedicine has facilitated remote healthcare delivery, enabling healthcare professionals to provide exceptional care from a distance. This is particularly relevant in the context of CKD and end-stage kidney disease (ESKD) patients, where the need for continuous care and monitoring is critical. This editorial underscored the escalating incidence of ESKD, driven by prevalent risk factors, such as diabetes, hypertension, and obesity, and the disparities in access to treatments among different populations. The integration of telemedicine in CKD and dialysis care presents a pathway toward a more accessible, efficient, and cost-effective healthcare delivery. It offers numerous benefits, including the convenience of remote monitoring, enhanced patient compliance, reduced healthcare costs, and improved patient satisfaction and quality of life. Telemedicine facilitates a multidisciplinary approach to care, allowing for timely intervention and follow-ups, which are crucial for patients undergoing dialysis. Moreover, the COVID-19 pandemic has accelerated the adoption of telemedicine, showcasing its effectiveness in maintaining continuity of care amid restrictions on patient contact. Despite its promising potential, its implementation of telemedicine faces several challenges, including regulatory hurdles, concerns about the security of medical information, and the adequacy of virtual platforms to capture crucial health indicators. In addition, the financial implications of telemedicine and its long-term sustainability remain areas requiring further investigation. In conclusion, telemedicine holds significant promise in enhancing the care and management of CKD and dialysis patients. It offers a vital solution to overcome the geographical barrier, improve access to care, and alleviate the strain on healthcare systems. However, further research is needed to fully understand its benefits compared to traditional care models and to address the challenges associated with implementation. The expansion of telemedicine in kidney care signifies a step toward a more inclusive, efficient, and patient-centered healthcare future.

## Editorial

With the advancement of technology and virtual networking, the world has become a global borderless hub, which has tremendous potential in the field of different sectors of healthcare and medicine. Telemedicine is one such technological advancement that has provided overburdened healthcare systems globally with a pathway into the future of managing diseases and health problems [1]. The role of telemedicine in several healthcare fields is constantly evolving with new systems replacing the conventional systems where physical presence, commute, and appointment availability all hinder the prognosis of a patient seeking healthcare advice or management [2]. There is an array of high-quality audio and visual platforms that are constantly being developed, which enable healthcare professionals to provide high-quality care from a distance [3]. Over the last few decades, advancements in information technology have integrated telemedicine into routine care across various medical fields, including cardiology, nephrology, ophthalmology, psychiatry, and emergency medicine. This integration enables healthcare providers to deliver high-quality care across a broad spectrum of specialties [3]. The hesitance in the use of telemedicine has greatly reduced since the recent COVID-19 pandemic. This has also been seen across several departments, including the nephrology department. One of the major care requirements of this department is the constant need for improvement of dialysis care. Therefore, in this review, our focus is on exploring the scintillating potential of telemedicine in enhancing dialysis care and other kidney diseases.

The impact of telemedicine on chronic kidney disease (CKD)

Patient with end-stage kidney disease (ESKD) requires dialysis or kidney transplants is a rapidly increasing trend around the world. The incidence of ESKD in the USA and high-income Southeast Asian countries is high in comparison with high-income European countries, Australia, and New Zealand. This is likely due to the higher incidence of CKD risk factors, such as diabetes, hypertension, and obesity [4]. The ESKD incidence in the USA alone has been on the incline since 2011 [5]. The burden of ESKD is largely felt in the African American population and ethnic minorities, additionally by disadvantaged groups who cannot afford the expenses of the current treatments, and sheds light on the inequities of treatment in the current healthcare system [6]. Moreover, a recent meta-analysis comparing mortality rates across different dialysis methods offers critical insights into the effectiveness of these treatments and their impact on patient survival [7]. Therefore, to navigate these evident problems, the role of telemedicine may be pivotal. 

Telemedicine: a pathway for the future of CKD and dialysis care

CKD is a complex condition that requires a high level of care and close follow-up monitoring regularly with a multidisciplinary team, surveillance of laboratory values, and long-term support by healthcare providers [8]. CKD has a tremendous financial burden on the healthcare systems in developed countries. A recent review highlighted that the costs per patient for those with CKD stages 4 and 5 range from $5367 to $53,186 USD and from $20,110 to $100,593 USD for those with ESKD requiring dialysis [9]. Some major problems in the treatment of kidney disease are the barriers to providing care, such as geographic locations and late referrals to nephrologists, which in turn delay diagnosis and initiation of dialysis [10]. However, with the recent devastation of the COVID-19 pandemic, healthcare institutions collaboratively sought to provide care without physical contact. This opened new avenues in the use of telemedicine across the healthcare system. Subsequently, pandemic-enforced solutions, such as care delivery through video-based communication, became widely accepted throughout the world [8]. 

There is compelling evidence that supports that the transition of dialysis care to telemedicine may potentially change the outcomes of a patient undergoing dialysis. One study assessing the role of telemedicine in peritoneal dialysis revealed that a well-structured protocol for dialysis care utilizing telemedicine has several benefits. Some of these benefits are assessment of a patient in a comfortable environment, compliance with prescribed techniques, observation and prevention of potential hazards that may cause infections, and reinforcement of patient confidence in care and encouragement [1]. A major problem in dialysis care is monitoring and observing complex care patients, such as those with pacemakers and implantable cardioverters, and a plethora of studies have revealed that this problem is economically and efficiently tackled with the use of telemedicine [11-13]. Furthermore, the fact that although there is no requirement for a physical center to assemble a multidisciplinary team, the use of virtual communication teams can assess and notify patients about the treatments and management much quicker than conventional appointment systems. This strategy not only combats the heavy costs to the healthcare systems but also lifts the financial burden from the patients themselves. Another key issue is follow-up appointments for observation and monitoring; this is usually hindered by patients’ reluctance to attend several appointments. This may be potentially facilitated by a communication-based follow-up and improve safety within the home setting, making it easier to choose and live with home dialysis [14].

Studies utilizing video-based telemedicine for both hemodialysis and peritoneal dialysis have shown promising results, with increased patient enrollment and reduced dropout rates [15-16]. Furthermore, these studies have also reported that there were fewer associated emergency visits and hospital admissions. In addition, patients reported a huge increase in confidence in healthcare, empowerment, and independence, and they stated an overall improvement in quality of life [17]. Since the COVID-19 pandemic, the increase in telemedicine has been rapid, and data show that in Ontario, Canada, alone, there was a 40% increase in the use of telemedicine from 2% prior to 2019 [18]. Video-based telemedicine shows great potential for patients in hospital or home-based dialysis patient to interact with relevant specialists quickly and implement the advice given by clinicians, and these will not only improve the clinical outcomes but also patient satisfaction. The economic benefits of telemedicine in dialysis care have limited data currently available. However, some studies have reported a decrease in healthcare costs due to a reduction in medical service use and limiting travel for house visits [19].

Limitations

Although the evidence of the positive outcomes of telemedicine in dialysis care is considerable, there are a few limitations that may occur with its implementation routinely. First, current laws and policies are not so detailed in the use of telemedicine; this opens doors to potential fraud and misuse of medical information. Therefore, policies will need to be reviewed in order to address these issues prior to routine implementation in dialysis care. Furthermore, the costs of service use may be reduced, but currently, there is insufficient data on the benefits of telemedicine financially. With the growth of telemedicine, it is inevitable that the costs of maintaining the virtual platforms will increase and that would negate the potential cost benefits that may be noted initially. Lastly, the routine use of virtual medicine may limit the physical exam to detail, which sometimes is essential in observing physical and mental health signs that may not be picked up due to the lack of human contact. 

Conclusions

Telemedicine is rapidly growing in the modern world of healthcare. This is also true for the treatment of kidney diseases and dialysis care. CKD and ESKD are complex diseases that are burdensome to both patients and healthcare providers. The use of telemedicine has opened new avenues for potential growth and improvement in dialysis care. Telemedicine has brought several beneficial aspects to modern dialysis care. Some benefits include higher patient satisfaction, easier follow-ups, reduced costs, improved quality of life, reduced burden on healthcare centers and providers, better prognosis, and comfortable environments to receive care for patients. The growth of telemedicine may increase patient compliance to follow advice and attend appointments easily. The potential of telemedicine is undoubtedly present and severely less explored by researchers. Therefore, the exact benefits of telemedicine in comparison with conventional care need to be analyzed, and therefore future research is warranted.
